# Antioxidant and Antidiabetic Activity of *Helicteres isora* (L.) Fruits

**DOI:** 10.4103/0250-474X.59557

**Published:** 2009

**Authors:** M. Suthar, G. S. Rathore, A. Pareek

**Affiliations:** L. B. S. College of Pharmacy, Udai Marg, Tilak Nagar, Jaipur-302 004, India; 1 L. M. College of Science and Technology (Pharmacy Wing), Sector-A, Shastri Nagar, Jodhpur-342 003, India

**Keywords:** Antioxidant activity, *Helicteres isora* (L.), isolated rat hemi-diaphragm model, thiobarbituric acid reactive species

## Abstract

The present investigations evaluated the antioxidant and antidiabetic activity of *Helicteres isora* (L.) fruits belonging to the family Sterculiaceae. The hot water extract of *Helicteres isora* fruits was prepared and screened for its in vitro antioxidant activity using 1,1-diphenyl,2-picryl hydrazyl assay, ß-carotene-linoleate model and microsomal lipid peroxidation or thiobarbituric acid reactive species assays and the IC_50_ values were calculated. Antidiabetic effect was studied using the in vitro glucose uptake in the isolated rat hemi-diaphragm model. The hot water extract of *Helicteres isora* showed maximum activity with IC_50_ value 25.12±0.18 μg/ml for 1,1-diphenyl,2-picryl hydrazyl assay method, and low activity with IC_50_ value 740.64±4.76 μg/ml for microsomal lipid peroxidation assay. In the ß-carotene-linoleate model, the extract showed 45.63% antioxidant activity. The extract produce a significant (P<0.05) uptake of glucose by isolated rat hemi-diaphragm but less effective to that of the reference drug, metformin. The hot water extract of fruit of *Helicteres isora* exhibited significant antioxidant activity and moderate antidiabetic activity and merits further investigation in animal models and isolation of its active constituents.

Diabetes mellitus (DM), a state of chronic hyperglycaemia, is a common disease affecting over 124 million individuals worldwide[1,2]. DM is associated with high risk of atherosclerosis, renal, nervous system and ocular damage[3]. Uncontrolled hyperglycemia appears to be the principal biochemical abnormality that underlies the increased oxidative load in DM. Increased oxidative stress may contribute to the pathogenesis of the diabetic complication. In addition, increased oxidative injury has been implicated in the premature age related changes in DM[4]. Multiple studies[5–9] have shown that type 2 diabetes is accompanied by increased oxidative damage to all bio-molecules, especially lipids. Results of studies in animal models and in humans have demonstrated that diabetes is associated with oxidative stress, which is exhibited by elevated blood levels of lipid per-oxidation products (markers of oxidative stress), especially associated with poor blood glucose control[10–15].

*Helicteres isora* L. is a large arborescent shrub of the family Sterculiaceae[16]. It is used as an antigastrospasmodic, anthelmintic, antispasmodic, antipyretic, antidiarrheal, antidysenteric[17] and as a tonic after childbirth[18]. Stems of this plant are used as anthelmintic, colic, and aphtha, while fruits are used as colic, anticonvulsant, and abdominalgia[19]. Traditionally, the root juice is claimed to be useful in diabetes, emphysema, and snakebite[20,21]. From the roots, betulic acid, daucosterol, sitosterol, isorin[22] were isolated. Cucurbitacin B and isocucurbitacin B were isolated and reported to possess cytotoxic activity[23]. In addition, Hattori and co-workers reported an inhibitory activity of the water extract of fruits of *H. isora* against reverse transcriptase from avian myeloblastosis virus[24] and antiHIV-1 activity[25]. Six neolignans, the helicterins A–F were isolated from aqueous extract of the fruits[26], plant also contains flavonoid glucosides[27]. The present study was undertaken to verify the claim and evaluate the antidiabetic and antioxidant property of the fruits of *H. isora*.

The fruits of *Helicteres isora* (L.) were collected from the local market of Ootacamund, in the month of June 2006 and authenticated at the Survey of Medicinal Plants and Collection unit, Ootacamund, Tamilnadu, India. 1,1-Diphenyl-2-picryl hydrazyl (DPPH) was obtained from Sigma Aldrich Co., St. Louis, USA. Ascorbic acid, rutin and α-tocopherol were obtained from Merck Ltd., Mumbai, India. Glucose kit was obtained from Randox, potassiumm ferricyanide, trichloroacetic acid, ferric chloride, dimethylsulfoxide (DMSO), thiobarbituric acid, sulphanilic acid, linoleic acid, Tween 40, hydrogen peroxide, glacial acetic acid, dibasic sodium hydrogen phosphate, sodium bicarbonate, magnesium chloride, calcium chloride, potassium chloride, sodium chloride were purchased from Ranbaxy Laboratories Ltd., Mohali, India and S. D. Fine Chem., Mumbai, India. All chemicals and solvents used were of analytical grade.

The collected fruits were shade dried, coarsely powdered and extracted with hot water by maceration process. The extract was filtered and concentrated by vacuum and kept in a vacuum desiccator for complete removal of solvent. Hot water extract of fruits of *Helicteres isora* was obtained in the yield of 1.8% w/w.

Hot water extract of fruits of *Helicteres isora* and the standard antioxidants (ascorbic acid, α-tocopherol and rutin) were dissolved in distilled DMSO separately and used for *in vitro* antioxidant assays using three different methods. The stock solutions were serially diluted with the respective solvents to obtained lower dilutions.

The extract was tested for its *in vitro* antioxidant activity using standard methods. In all these methods, a particular concentration of the extract or standard solution was used which gave final concentration of 1000 to 0.45 μg/ml after all the reagents were added. Absorbance was measured against a blank solution containing the extract or standards. A control test was performed without the extracts or standard. Percentage scavenging and IC_50_ values±SEM (IC_50_ value is the concentration of the sample required to inhibit 50% of radical) were calculated.

The assay was carried out in a 96 well micro titre plate. To 200 μl of DPPH solution, 10 μl of each of the test sample or standard solutions were added separately in wells of the micro titre plate. The final concentration of the test and standard solutions used were 1000 to 1.95 μg/ml. The plates were incubated at 37° for 20 min and the absorbance of each solution was measured at 490 nm, using an ELISA reader (Bio-Rad Laboratories Inc, California, USA, Model 550)[28].

The antioxidant activity of extract was evaluated by the β-carotene-linoleate model system[29]. A solution of β-carotene was prepared by dissolving 2 mg of β-carotene in the 10 ml of chloroform. This solution (2 ml) was pipetted into a 100 ml round bottom flask. After chloroform was removed under vacuum, 40 mg of purified linoleic acid, 400 mg of Tween 40 emulsifier, and 100 ml of aerated distilled water were added to the flask with vigorous shaking. Aliquots (4.8 ml) of this emulsion were transferred into test tubes containing different concentrations of the extracts (0.2 ml). As soon as the emulsion was added to each tube, the zero time absorbance was measured at the 470 nm using a spectrophotometer. The tubes were placed at 50° in a water bath, and measurement of absorbance was recorded after 2 h; a blank, devoid of β-carotene, was prepared for background subtraction. The same procedure was repeated with the ascorbic acid, as a positive control. Antioxidant activity was calculated using the following equation: antioxidant activity = (β-carotene content after 2 h of assay/initial β-carotene content)×100

The test samples (100 μl) of different concentrations were added to 1 ml of liposome mixture, control was without test sample. Lipid peroxidation was induced by adding 10 μl FeCl_3_ (400 mM) and 10 μl L-ascorbic acid (200 mM). After incubation for 1 h at 37°, the reaction was stopped by adding 2 ml of 0.25 N HCl containing 15% trichloroacetic acid and 0.38% thiobarbituric acid and the reaction mixture was boiled for 15 min then cooled, centrifuged and absorbance of the supernatant was measured at 532 nm[30].

Wistar rats of either sex weighing between 160-180 g were selected. The animals maintained on a standard pellet diet (water *ad libitum*), and fasted over night were used. The animals were killed by decapitation and diaphragms were dissected out quickly with minimal trauma and divided into two halves. The hemi diaphragms were then rinsed in cold Tyrode solution (without glucose) to remove any blood clots and were placed in small culture tubes containing 2 ml Tyrode solution with 2% glucose and incubated for 30 min at 370 nm in an atmosphere of 100% O_2_ with shaking[31,32].

Six groups containing five numbers of graduated test tubes (n=5) each, were taken as given in Table 1. Two diaphragms from the same animal were not used for the same set of experiment. Following incubation, the hemi diaphragms were taken out and weighed. The glucose content of the incubated medium was measured by glucose oxidase-peroxidase (GOD-POD) method. The uptake of glucose was calculated in mg/g of moist tissue/30 min. Glucose uptake per gram of tissue was calculated as the difference between the initial and final glucose content in the incubated medium.

**TABLE 1:** EFFECT OF HOT WATER EXTRACT OF *HELICTERES ISORA* (L.) FRUITS ON GLUCOSE UPTAKE BY ISOLATED RAT HEMI DIAPHRAGM

S. No.	Incubation Medium	Glucose Uptake (mg/g/30 m)
Group 1	2 ml Tyrode solution with 2 % glucose (Control group)	3.46±0.21
Group 2	2 ml Tyrode solution with 2 % glucose+0.62 ml insulin (0.4 IU/ml)	8.76±0.26*
Group 3	2 ml Tyrode solution with 2 % glucose+ 0.4 ml metformin (1 mg/ml)	16.17±0.20*
Group 4	2 ml Tyrode solution with 2 % glucose+0.62 ml insulin (0.4 IU/ml)+0.4 ml metformin (1 mg/ml)	17.71±0.48*
Group 5	Tyrode solution with 2 % glucose+1.0 ml of *H. isora* extract (2 mg/ml)	4.85±0.25*
Group 6	Tyrode solution with 2 % glucose+1.0 ml of *H. isora* extract (2 mg/ml)+0.62 ml insulin (0.4 IU/ml)	8.94±0.49*

Values are mean±SEM, n=5

* P<0.05 as compared to control, The total volumes for all the groups were made up to 4 ml with distilled water.

Glucose is determined after enzymatic oxidation in the presence of GOD. The hydrogen peroxide formed reacts, under catalytic influence of POD, with phenol and 4-aminophenazone to form a red-violet quinoneimine dye as indicator. This method is used for quantitative *in vitro* determination of glucose content in various samples. Ten microlitres of sample and 1 ml reagent were mixed together and incubated for 25 min at 15-25° or 10 min at 37°. Absorbance of standard (A_standard_) and sample (A_sample_) were measured against the reagent blank within 60 min. The time interval from sample addition to read time must be exactly the same for standard/control and sample. Glucose concentration (mmol/l)= (A_sample_/A_standard-_)×55.5 or (mg/dl)= (A_sample_/A_standard-_)×100. Results were represented as mean±SEM. Statistical analysis was performed by one-way ANOVA followed by Dunnett's test. P values <0.05 were considered significant.

The preliminary phytochemical investigation of the extract revealed the presence of steroids, terpenoids, alkaloids, carbohydrate and phenolic compounds such as tannins, and flavonoid. These compounds are likely to be responsible for the observed antioxidant and antidiabetic activity of this extract either single or in synergy with one another.

*Helicteres isora* hot water extract was tested for its antioxidant activity in three different *in vitro* models. The antioxidant activities measured are given in Table 2. *Helicteres isora* showed IC_50_ value 25.12±0.18 μg/ml for DPPH method, which was comparable to that of ascorbic acid (IC_50_ =2.75±0.29 μg/ml) and rutin (IC_50_ =7.89±0.51 μg/ml). DPPH is a free radical, stable at room temperature, which produces a purple colour solution in methanol. It is reduced in the presence of an antioxidant molecule, giving rise to uncoloured methanol solution. In lipid peroxidation method, IC_50_ value was found to be 740.64±4.76 μg/ml, which compares favourably with α-tocopherol (IC_50_=91.17±0.24 μg/ml). The antioxidant activity of extract was estimated by bleaching of β-carotene as model systems. The controls (no additive) are decolorized within few min, indicating that rapid oxidation occurred. The addition of extract and ascorbic acid enhanced the bleaching time of β-carotene. In β-carotene-linoleate model system, percent antioxidant activity of extract was found to be 45.63%, which is very good when compared to ascorbic acid (91.65%). The IC_50_ values obtained, however, for the extract in two methods were found to be higher than the standard used, indicating its low activity compared to the standards.

**TABLE 2:** ANTIOXIDANT ACTIVITY OF HOT WATER EXTRACT OF *HELICTERES ISORA* (L.) FRUITS

Extract/Standard	IC_50_ values±SEM* (μg/ml)		% Antioxidant activity ß-carotene linoleate
	
	DPPH	TBARS	
Hot water extract	25.12±0.18	740.64±4.76	45.63%
Ascorbic acid	2.75±0.29	-	91.65%
Rutin	7.89±0.51	-	-
α- tocopherol	-	91.17±0.24	-

* Average of three independent determinations, three replicates, and values are mean±SEM. IC_50_=Concentration of the sample/standard required to inhibit 50% of free radicals, DPPH=1,1-diphenyl-2-picryl hydrazyl, TBARS=Thiobarbituric acid reactive species, SEM=Standard error mean.

The isolated diaphragm of the rat is a suitable tool for the experimental study of the glucose uptake and glycogen synthesis by muscle tissue, and demonstrated that these processes are stimulated by *in vitro* addition of insulin many investigators have used the method to study the hormonal control of carbohydrate metabolism[33].

Levine and Goldstein proposed that the uptake of glucose by muscle such as diaphragm occurs in two stages, the rate-limiting penetration of the muscle cell by glucose and metabolism of glucose within the cell. It is the penetration of the muscle cell by glucose which is the rate-limiting step in the uptake of glucose by isolated diaphragm and that insulin or factors which inhibit oxidative phosphorylation augment glucose uptake by increasing penetration[34].

The antidiabetic activities measured are given in Table 1. Hot water extract of fruits of *Helicteres isora* enhances the uptake of glucose by isolated rat hemi-diaphragm (P<0.01) but less effective than insulin. *Helicteres isora* extract showed following results: control 3.46±0.21, HI (500 μg/ml) 4.85±0.25, insulin 8.76±0.26 and metformin (100 μg/ml) 16.17±0.20 in mg/g/30 min. and, addition of insulin with extract was also not showed any remarkable increase in glucose uptake; result was 8.94 ± 0.49 mg/g/30 min. It showed that *Helicteres isora* extract have moderate effect on glucose uptake by isolated rat hemidiaphragm.

The results obtained in the present study clearly demonstrate that the hot water extract of *Helicteres isora*, which can effectively scavenge various reactive oxygen species/free radicals under in vitro conditions. This may be due to the number of stable oxidized products that it can form after oxidation or radical scavenging. The broad range of activity of the hot water extract of *Helicteres isora* suggests that multiple mechanisms are responsible for the antioxidant and antidiabetic activity.
